# Mutation Load of Single, Large-Scale Deletions of mtDNA in Mitotic and Postmitotic Tissues

**DOI:** 10.3389/fgene.2020.547638

**Published:** 2020-10-02

**Authors:** Tina D. Jeppesen, Morten Duno, John Vissing

**Affiliations:** ^1^Copenhagen Neuromuscular Center, Rigshospitalet, University of Copenhagen, Copenhagen, Denmark; ^2^Department of Clinical Genetics, Rigshospitalet, University of Copenhagen, Copenhagen, Denmark

**Keywords:** mtDNA mutation, mtDNA deletion, phenotype–genotype, mitochondrial myopathy, CPEO, chronic progressive external ophthalmoplegia

## Abstract

It is generally accepted that patients with chronic progressive ophthalmoplegia caused by single large-scale deletion (SLD) of mitochondrial DNA (mtDNA) only harbor mutation in skeletal and eye muscles. The aim of this study was to investigate the presence and the level of heteroplasmy of mtDNA deletions in mitotic tissues of patients displaying mtDNA deletion of mitotic tissues in patients with SLDs and pure muscle phenotype. MtDNA mutation load was studied in three mitotic (urine epithelial cells, buccal mucosa, and blood) and one postmitotic (skeletal muscle) tissues in 17 patients with SLDs of mtDNA and pure muscle involvement. All patients had mtDNA deletion in skeletal muscle, and 78% of the patients also displayed the mtDNA deletion in mitotic tissues. The mtDNA mutation load was higher in skeletal muscle versus mitotic tissues. The mtDNA mutation load did not correlate with age of sampling of tissues, but there was a correlation between the mtDNA mutations load in skeletal muscle and (1) the site of 5′ end breaking point of the SLD, (2) the size of SLD, (3) the number of affected tRNAs, and (4) age at onset (*r* > 0.58, *P* < 0.05). The findings indicate that mtDNA mutation in mitotic tissue is common in patients with SLDs of mtDNA. The lack of correlation between age of tissue sampling, age at onset, and mtDNA mutation load in mitotic tissues indicates that there is no extensive post-natal modification of mtDNA mutation load in mitotic tissues of patients with pure muscle phenotype.

## Introduction

Single, large-scale deletion (SLDs) of mitochondrial DNA (mtDNA) is the most common sporadic, pathogenic mtDNA aberration (Chinnery and Turnbull, 2000). SLD can occur at any point of life, but those SLDs of mtDNA that lead to primary mitochondrial disease are thought to occur during embryogenesis. Patients can present with highly variable phenotypes from pure muscle affection [myopathy and chronic progressive external ophthalmoplegia (CPEO)], to multiorgan involvement, i.e., Kearns–Sayre (KSS) and Pearson syndromes (PS). This variability in phenotypes may in part be explained by differences in the level of mtDNA mutation in different tissues among patients. The mechanism behind this variability is unknown, but may be a consequence of prenatal or postnatal modification of mtDNA mutation load in different tissues, depending on the mitotic status of the tissues (McShane et al., 1991), the specific mutation type (López-Gallardo et al., 2009) especially when complex-specific protein-encoding genes are deleted (Rocha et al., 2018). To investigate the occurrence of mutated mtDNA in mitotic tissue in patients with SLD of mtDNA and pure muscle phenotype, we studied the presence of SLDs of mtDNA in both mitotic and postmitotic tissues in 17 patients, aged 20–77 years, and tested if it was associated with age of patients at tissue sampling, age at onset, size of deletion, and number of affected tRNAs.

## Materials and Methods

The study was approved by the Scientific Ethical Committee of Copenhagen (no. KF 01-075/00). The subjects were all informed about the nature and risks of the study and gave written consent to participate.

### Subjects

The cohort consisted of 17 patients (seven women) with sporadic SLDs of mtDNA and pure muscle affection (Table 1).

**TABLE 1:** Genetic, clinical and demographic findings in 17 patients with single large-scale deletion of mtDNA.

**Patient**	**Single large-scale deletion**		**Affected genes**	**Gender**	**Age at onset**	**Age biopsy**	**Clinical symptoms**	**mtDNA mutation load (%)**			
	**Site**	**Size (bp)**	**Number**					**M**	**B**	**UEC**	**BM**
#1	8373–16071	7,698	10	M	62	65	EI	10	5	5	5
#2	5793–11129	5,336	6	F	36	45	EI, CPEO	29	NP	NP	NP
#3	8483–13459	4,976	5	M	53	61	EI, CPEO	35	NP	4	NP
#4	8580–11531	2,951	2	F	46	77	EI, CPEO	64	NP	NP	NP
#5	13585–15596	2,011	1	M	25	41	EI, CPEO	73	5	3	5
#6	6098–12201	6,103	5	F	39	50	CPEO	9	1	3	NP
#7	11290–15912	4,622	7	F	30	64	EI, CPEO	70	NP	5	5
#8	9110–14605	5,495	5	F	31	63	EI, CPEO	61	NP	NP	9
#9	6391–13186	6,795	7	M	18	33	EI, CPEO	33	7	NP	5
#10	7177–13767	6,590	7	M	22	46	EI, CPEO	51	NP	NP	3
#11	12113–14422	2,309	3	F	18	57	EI, CPEO	77	NP	NP	NP
#12	10383–12881	2,498	4	M	15	40	EI, CPEO	70	17	5	5
#13	8937–13030	4,093	6	M	17	20	EI, CPEO	63	5	5	5
#14	8483–13459	4,976	5	M	28	38	EI, CPEO	58	8	3	NA
#15	8111–14548	6,437	6	F	57	60	CPEO	20	5	5	5
#16	8577–12983	4,406	6	M	33	39	CPEO, DM	16	10	10	10
#17	8637–16073	7,436	10	M	15	24	EI, CPEO	32	8	85	5

*F, female; M, male; EI, exercise intolerance; CPEO, chronic progressive external ophthalmoplegia; DM, diabetes mellitus type II; B, blood; M, skeletal muscle; UEC, urine epithelial cells; BM, Buccal mucosa; Age biopsy, age of the patient at time of tissue sampling; Age at onset, age of the patient at onset of symptoms; NP, Not Present (large-scale deletion of mtDNA is not detectable).*

### Tissue Sampling

In each subject, a sample was taken from blood, buccal mucosa, urine epithelial cells (UECs), and skeletal muscle. All tissue samples were collected on the same day. A 5 mL blood sample was obtained in a syringe containing ethylenediaminetetraacetic acid. Sample for buccal mucosa cells was obtained by brushing each cheek in the oral cavity with a cytobrush for 30 s, and UEC was gathered after a spun of 20 mL of urine and resuspended in Tris buffer. All samples were stored at 5°C until DNA isolation. The needle muscle biopsy was performed in the vastus lateralis muscle, and the muscle biopsy was frozen immediately after sampling in isopentane, cooled by liquid nitrogen, and placed at −80°C until genetic analysis.

### Molecular Genetic Analysis

DNA from blood, buccal mucosa, UECs, and skeletal muscle was extracted using the QIAamp^®^ DNA Mini Kit (Qiagen GmbH, Hilden, Germany), according to the manufacturer’s protocol for the different tissues. The deletion breakpoints were identified by Sanger sequencing of polymerase chain reaction (PCR) fragments generated from the muscle-derived mtDNA and specified according to the revised Cambridge reference sequence (AC J01415) (Anderson et al., 1981). Quantification of the individual deletions was performed by real-time PCR essentially as described by He et al. (2002), using additional primers targeting the deleted proportion of the mtDNA (Supplementary Figure 1). As the comparison is relative, the normal sample determines the sensitivity; that is the variation between the two real-time PCR reactions on normal samples. It should be noted that the overall variation is approximately 3–5%, and thus, mtDNA mutation levels below 10% may therefore be up to 100% inaccurate.

The standard curves of the three quantitative PCR (qPCR) reactions used for quantifying the different deletions are shown in Supplementary Figure 1. The ND1 target was identical to one originally described by He et al. (2002), and the ND3 and ND5 targets were designed for the present study and target all the identified deletions. The standard curves are generated from a 10 × dilution series.

Individual deletion-specific PCR reactions were designed using primers flanking the individual breakpoints, giving rise to 200–300 bp deletion-specific PCR products assessed by gel electrophoresis.

In samples where mtDNA deletion could not be verified by qPCR, we performed a deletion-specific PCR. If the deletion-specific PCR could not confirm the presence of the SLD in the sample, the lack of mtDNA mutation load was notes as “NP” (not present) in Table 1.

### Statistical Analysis

Statistical analysis was calculated using Systat software. Spearman rank coefficients were calculated for correlations between mutation load in the different cell types and for the correlations among tissue mutation loads and age of patient at time of testing and age at onset. A one-way analysis of variance was applied for comparing degrees of heteroplasmy among tissues using multiple comparison estimation (Bonferroni correction). Scatterplots with regression analysis are presented. A significance level of 0.05 or less was considered statistically significant. All values are expressed as mean ± SE, except for the data in Table 1.

## Results

**FIGURE 1:**
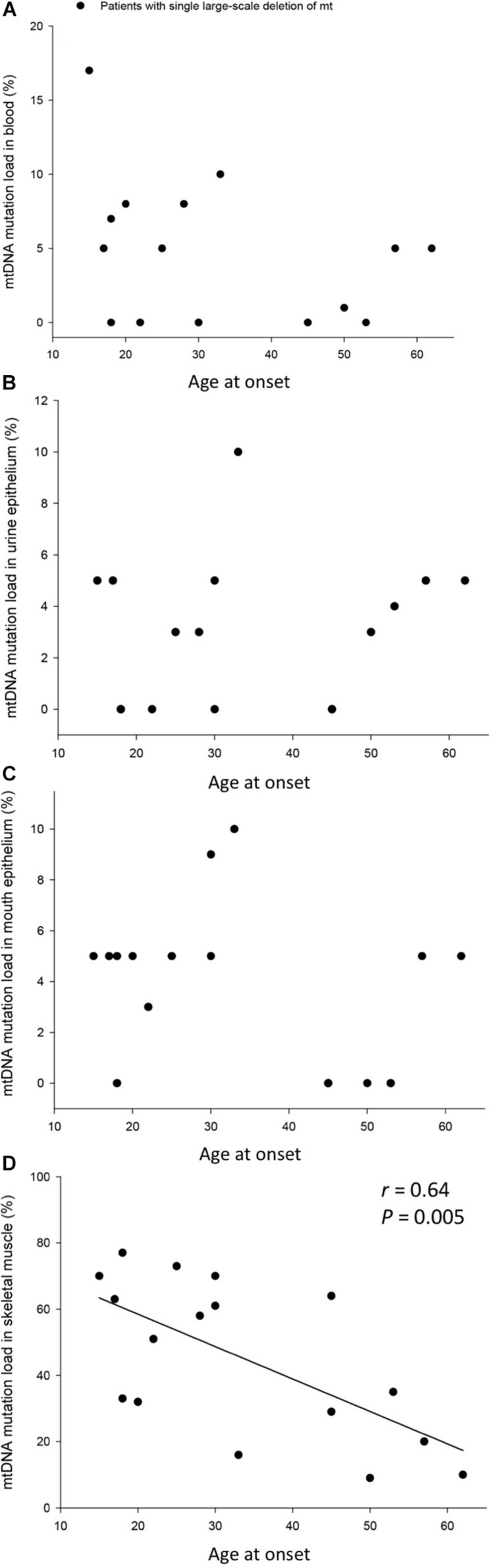
Correlation between age at onset of symptoms and percentage mitochondrial DNA (mtDNA) mutation load in blood **(A)**, urine epithelium **(B)**, mouth epithelium **(C)**, and skeletal muscle **(D)** in the individual 17 patients with single large-scale deletion of mtDNA. In graph **(B)**, patient 17 was left out in order not to have a ceiling effect.

**FIGURE 2:**
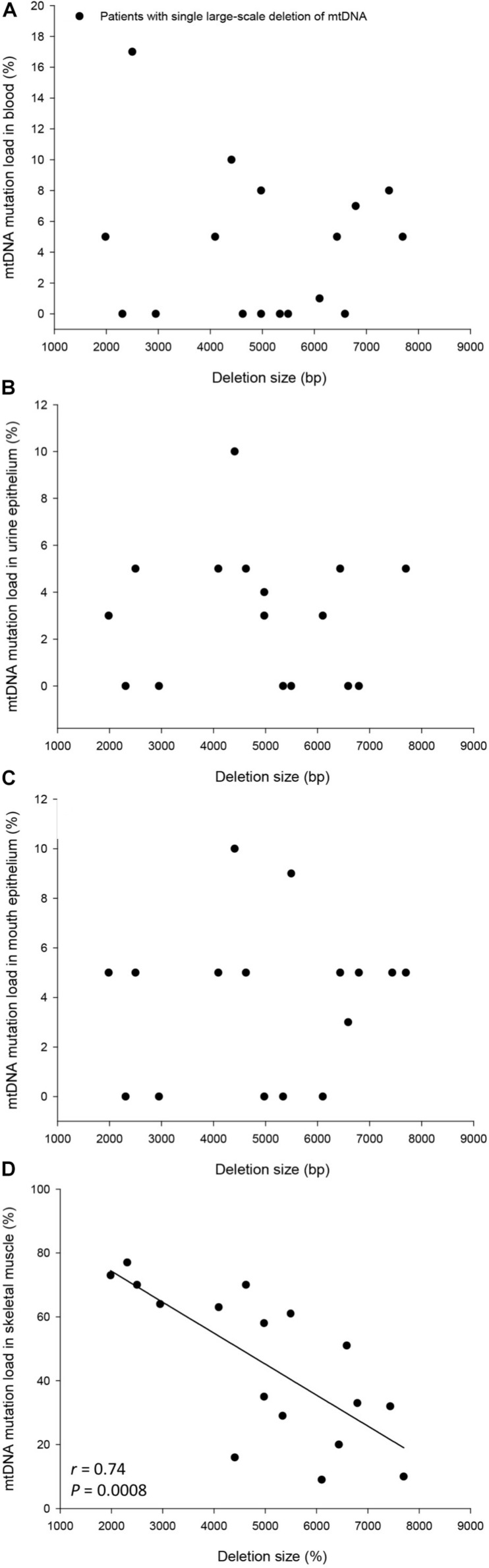
Correlation between size of deletion and percentage mitochondrial DNA (mtDNA) mutation load in blood **(A)**, urine epithelium **(B)**, mouth epithelium **(C)**, and skeletal muscle **(D)** in the individual 17 patients with single large-scale deletion of mtDNA. In graph **(B)**, patient 17 was left out in order not to have a ceiling effect.

**FIGURE 3:**
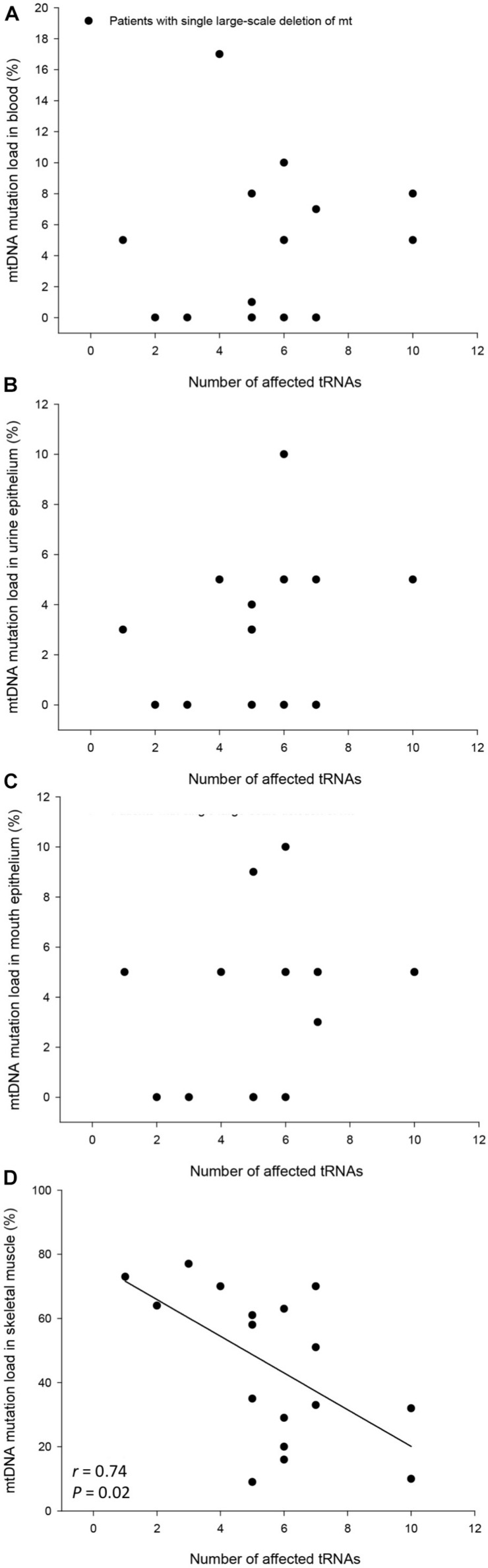
Correlation between number of affected tRNAs and percentage mitochondrial DNA (mtDNA) mutation load in blood **(A)**, urine epithelium **(B)**, mouth epithelium **(C)** and skeletal muscle **(D)** in the individual 17 patients with single large-scale deletion of mtDNA. In graph **(B)** patient number #17 was left out in order not to have a ceiling effect.

The mtDNA mutation loads were determined in the patients with SLD in blood (mesoderm, 4 ± 1%), skeletal muscle (mesoderm, 45 ± 6%), buccal mucosa (ectoderm, 4 ± 1%), and UECs (endoderm, 8 ± 5%) and was lower in mitotic versus postmitotic tissue (*P* < 0.05). There was no correlation between mutation load found in the four tissues of the patients and age of the patients at tissue sampling (Supplementary Figure 2). There was no correlation between individual levels of mtDNA found in the four tissues tested in the patients with SLD (Supplementary Figures 3,4). There was an inverse correlation between age at onset of symptoms and mtDNA mutation load in skeletal muscle (*r* = 0.64, *P* = 0.005), whereas age at onset of symptoms did not correlate with mtDNA mutation load found in the mitotic tissues of the patients with SLD (Figure 1). There was an inverse correlation between mtDNA mutation load in skeletal muscle and the size of mtDNA mutation load in skeletal muscle (*r* = 0.76, *P* = 0.0004; Figure 2), whereas there was no correlation between mtDNA mutation load found in mitotic tissue of the patients with SLD and the size of mtDNA mutation of patients with SLD (Figure 2). There was an inverse correlation between mtDNA mutation load in skeletal muscle and the number of tRNAs that were affected (*r* = 0.74, *P* = 0.02; Figure 3), whereas the number of tRNAs affected did not correlate with the mtDNA mutation load found in mitotic tissue of the patients with SLD (Figure 3). Age at onset of symptoms in patients with SLD did not correlate with the size of deletion, site/start of deletion, and number of tRNAs (Supplementary Figure 5).

## Discussion

In the present study, we investigated the mtDNA mutation level in mitotic and postmitotic tissues from the three germ layers in 17 patients with SLDs of mtDNA and pure muscle involvement. Two-thirds of the patients had SLDs in mitotic tissues. There was no correlation between age at tissue sampling, age at onset of symptoms, and the level of mtDNA mutation in mitotic tissues, which indicate that there had been no substantial postnatal modification of the level of mtDNA in mitotic tissue in these patients. Instead, there was a correlation between mtDNA mutation load in skeletal muscle and the size of deletion and number of affected tRNAs, which indicates that the size of SLD affects the modification of mutation load.

It has been proposed that the high variability in mtDNA mutation load among tissues in patients with SLD is a result of postnatal segregation. This idea has been supported by findings of increments in mtDNA mutation load in skeletal muscle of patients with KSS (Larsson et al., 1990) and extreme selection against mtDNA mutation in bone marrow of patients with PS (McShane et al., 1991). In these patients, bone marrow function shows improvement over time (McShane et al., 1991; Akman et al., 2004). In line with this, patient 17 in the present study with PS converted into pure muscle involvement at age 15 years. This patient presented with aplastic anemia and leukopenia at the age of 2 years. The patient was described with hypoactivity, but with no other obvious clinical abnormality or other organ manifestation. The patient received blood transfusions once to twice a month until the age of 5 years and less frequent until the age of 8 years where the patient no longer needed blood transfusions. At the age of 5 years, the patient developed hearing impairment and lost his hearing at the left ear at the age of 8 years. At the age of 14 years, the patient had converted into a pure muscle phenotype with ptosis bilaterally and incomplete external ophthalmoplegia and was diagnosed with mitochondrial disorder, which revealed a sporadic SLD of mtDNA genetic (m.8637-16073del). Genetic testing ruled out duplication. Cases such as this has prompted the idea that mtDNA mutation load is modified in tissues after birth in patients with SLD (McShane et al., 1991). However, the lack of correlation between age of patients at tissue sampling and the presence of mtDNA mutation in mitotic tissue indicate that a substantial time-related change in mtDNA mutation does not occur in mitotic tissues in patients with SLD and pure muscle involvement. Based on SLD being just as frequently present in young as older patients, this alone argues against a substantial postnatal shift of SLD in patients with SLD and pure muscle affection.

The most common deletion that is found in patients with SLD of mtDNA is a specific deletion encompassing 4,977 bp. Nevertheless, the SLD of mtDNA and thus the number of genes encoding both tRNAs and structural mRNAs that are affected vary tremendously. Many studies have investigated if size of deletion and number of affected tRNAs could be a determining factor for disease progression and severity, and results have been equivocal. In the present study, we found a direct correlation between the 5′ breakpoint, the size of the mtDNA deletions, and the mutation load in muscle, which has previously been shown in a meta-analysis on 149 patients and two other studies (Yamashita et al., 2008; López-Gallardo et al., 2009; Grady et al., 2014). These findings indicate that the mtDNA sequence impacts on the modification of the mtDNA mutation level.

Because the D-loop is essential for mtDNA maintenance, and correspondingly, a breakpoint positioned further downstream of the mtDNA will result in smaller mtDNA deletions; the finding of an inverse correlation between the level of mtDNA mutation load in muscle and the size of the SLD and number of affected tRNA genes indicate that the loss of function due to a smaller mtDNA deletion is less harmful than larger deletions of mtDNA. This finding indicates that the determining factor for cell survival is the amount of SLD of mtDNA rather than the specific sequence *per se*. This finding stands in contrast to data from animal studies, where smaller mtDNA fragments are prone to replicate more than larger fragments (Solignac et al., 1987). This mechanism could explain differences in phenotype expression between patients with KSS versus CPEO, where location and size of the SLDs often differ (López-Gallardo et al., 2009) and explain genotype-specific differences in mtDNA mutation load among tissues in patients with point mutations of mtDNA.

There is no consensus whether mtDNA mutation load of SLD correlates with phenotype and disease severity or not. Age at onset of symptoms has been used as an indirect measure of disease severity and prognosis. Although some studies have found a close relationship between age at onset of symptoms and mtDNA mutation load in skeletal muscle of patients with SLD and pure muscle involvement (Yamashita et al., 2008; Grady et al., 2014), others have not been able to conform this (López-Gallardo et al., 2009). In the present study, there was a direct correlation between age at onset and mtDNA mutation load in skeletal muscle, but no association between age at onset and size, mtDNA deletion start, and number of affected tRNAs. This finding indicates that mtDNA mutation load is still a strong denominator of disease progression in postmitotic tissue. The reason for a discrepancy among studies may be that determination of age at onset retrospectively can be difficult, because data rely on subjective experience and memory of symptom that may have developed years prior to diagnostic workup.

It has been proposed that patients with deletions covering genes in mtDNA coding region for cytochrome b, one of the three mtDNA encoding regions to cytochrome C (CO1–CO3) and/or encoding regions for complex V, have significantly earlier age at onset (Yamashita et al., 2008) of symptoms. Moreover, for the first time, Rocha et al. demonstrated that when associating the biochemical defect on single muscle fiber level, the nature of the deletion, i.e., the number of protein-encoding genes and the complexes affected by the deletion, had a high impact on the disease severity. In the present study, we did not perform single muscle fiber analysis, and perhaps for this reason, we did not find an association between affected/deleted protein-encoding genes and age at onset and mtDNA mutation load in either postmitotic or mitotic tissues.

In conclusion, our study showed that, in patients with SLDs and pure muscle phenotype who are generally associated with pure muscle affection, mtDNA mutation in mitotic tissues is a common finding. Moreover, the lack of a relationship between age of the patients and mtDNA mutation load in mitotic tissues indicates that the large range in mtDNA mutation load among mitotic tissues is not a result of an extreme selection for or against mutation. Lastly, the study indicates that the quantitative amount of mutated mtDNA is a determining factor for cell survival.

## Data Availability Statement

The raw data supporting the conclusions of this article will be made available by the authors, without undue reservation, to any qualified researcher.

## Ethics Statement

The studies involving human participants were reviewed and approved by the Scientific Ethical Committee of Copenhagen (No. KF 01-075/00). The patients/participants provided their written informed consent to participate in this study.

## Author Contributions

TJ and JV: study and design. TJ and MD: conduction of the study. TJ, JV, and MD: statistical analysis. TJ: writing the manuscript. JV and MD: critical revision of the manuscript. All authors contributed to the article and approved the submitted version.

## Conflict of Interest

The authors declare that the research was conducted in the absence of any commercial or financial relationships that could be construed as a potential conflict of interest.

## Supplementary Material

Supplementary Figure 1 Standard curves of the three qPCR reactions used for quantifying the different deletions.

Supplementary Figure 2 Correlation between Age of patients at tissue sampling versus percentage mitochondrial DNA (mtDNA) mutation load in blood **(A)**, urine epithelium **(B)**, mouth epithelium **(C)**, and skeletal muscle **(D)** in the individual 17 patients with single large-scale deletion of mtDNA. In graph **(B)** patient number #17 was left out in order not to have a ceiling effect.

Supplementary Figure 3 Correlation between mtDNA mutation load in skeletal muscle versus percentage mitochondrial DNA (mtDNA) mutation load in blood **(A)**, urine epithelium **(B)**, and mouth epithelium **(C)** in the individual 17 patients with single large-scale deletion of mtDNA.

Supplementary Figure 4 Correlation between percentage mitochondrial DNA (mtDNA) mutation load in blood versus mtDNA mutation load in urine epithelium **(A)**, percentage mitochondrial DNA (mtDNA) mutation load in blood versus mtDNA mutation load in mouth epithelium **(B)** and percentage mtDNA mutation load in urine epithelium versus mtDNA mutation load in mouth epithelium **(C)** in the individual 17 patients with single large-scale deletion of mtDNA. In graph **(A)** patient number #17 was left out in order not to have a ceiling effect and used as an example to show the difference from graph **(C)** where patient #17 has been left in.

Supplementary Figure 5 Correlation between Age at onset versus deletion size **(A)**, start of deletion **(B)** and number of tRNAs affected **(C)** in the individual 17 patients with single large-scale deletion of mtDNA.
