# Supplementary figures and images for: Mutation Load of Single, Large-Scale Deletions of mtDNA in Mitotic and Postmitotic Tissues
Source: Front Genet. 2020 Oct 2;11:547638. doi: 10.3389/fgene.2020.547638 (PMC7566915; doi:10.3389/fgene.2020.547638)

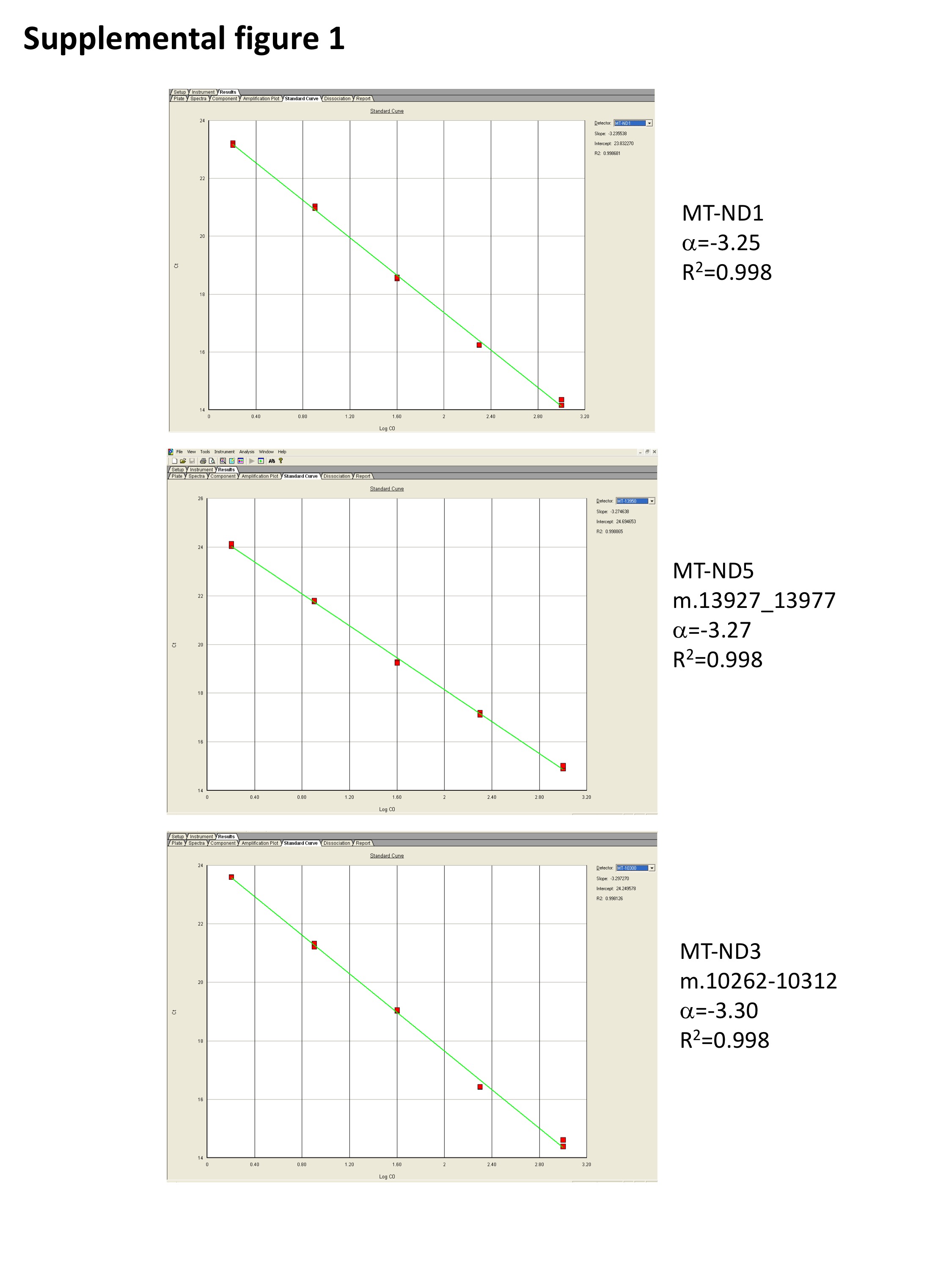

Supplement: Supplementary Figure 1 — Standard curves of the three qPCR reactions used for quantifying the different deletions. [file Image_1.JPEG]

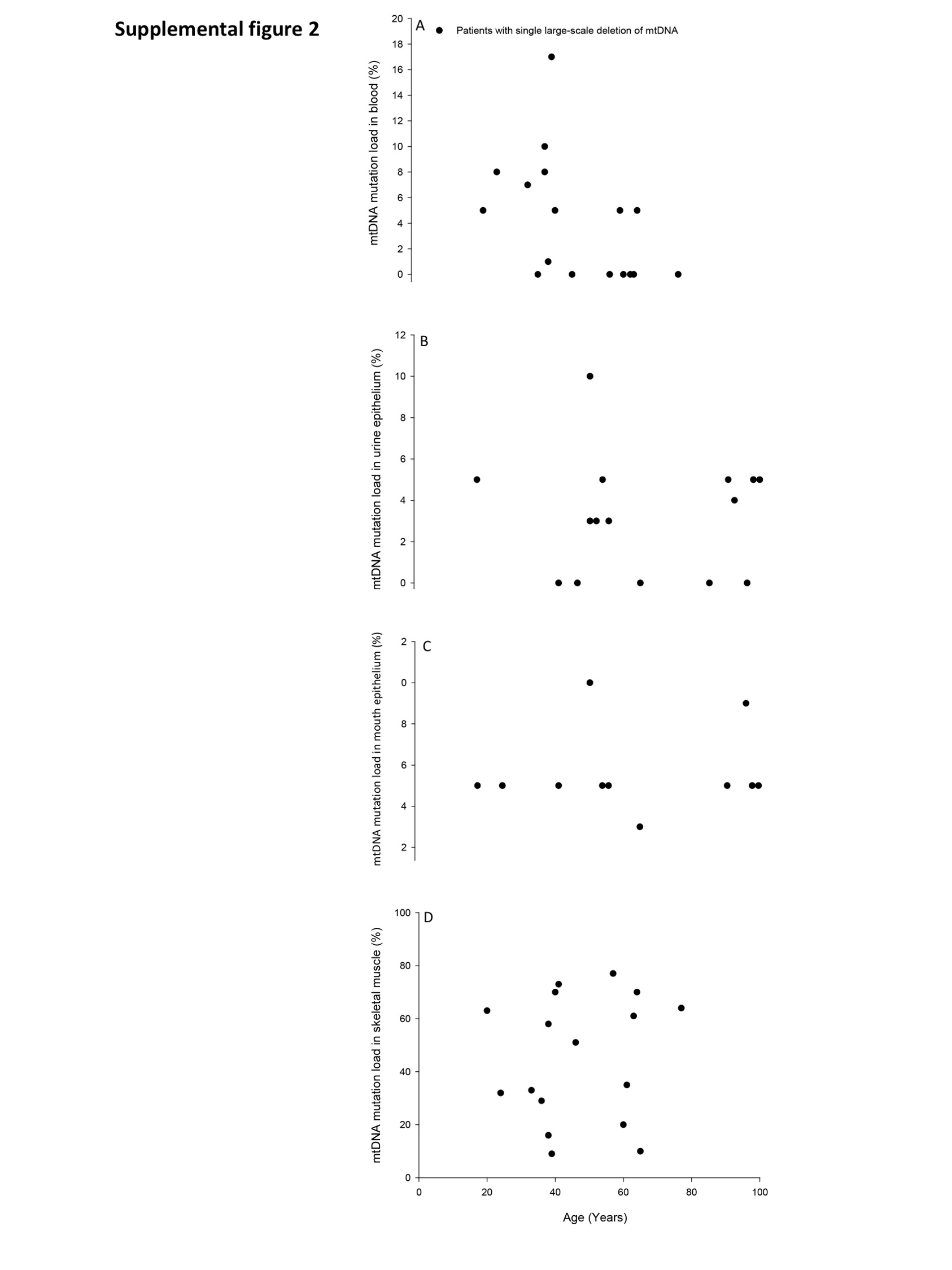

Supplement: Supplementary Figure 2 — Correlation between Age of patients at tissue sampling versus percentage mitochondrial DNA (mtDNA) mutation load in blood (A), urine epithelium (B), mouth epithelium (C), and skeletal muscle (D) in the individual 17 patients with single large-scale deletion of mtDNA. In graph (B) patient number #17 was left out in order not to have a ceiling effect. [file Image_2.JPEG]

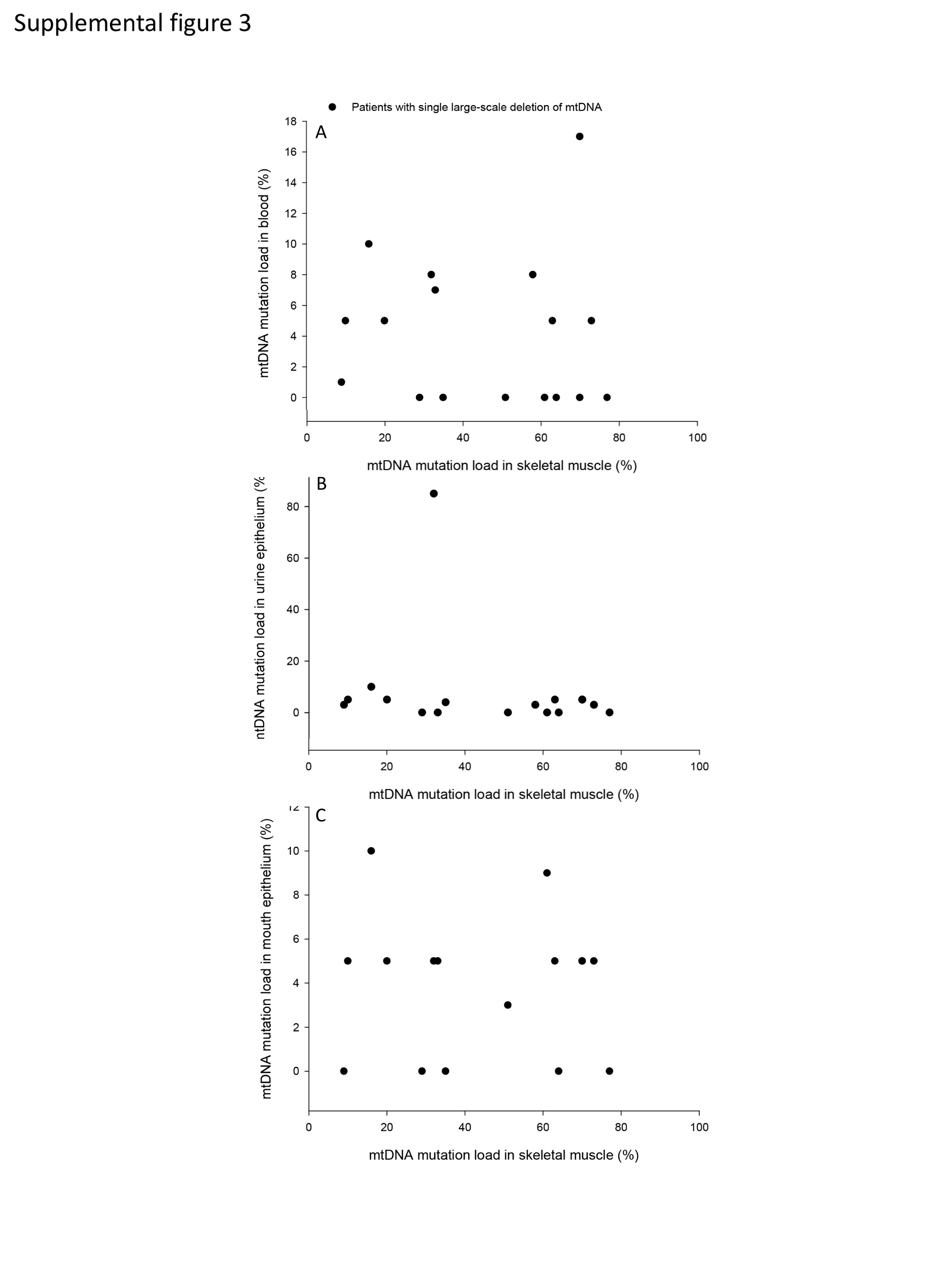

Supplement: Supplementary Figure 3 — Correlation between mtDNA mutation load in skeletal muscle versus percentage mitochondrial DNA (mtDNA) mutation load in blood (A), urine epithelium (B), and mouth epithelium (C) in the individual 17 patients with single large-scale deletion of mtDNA. [file Image_3.JPEG]

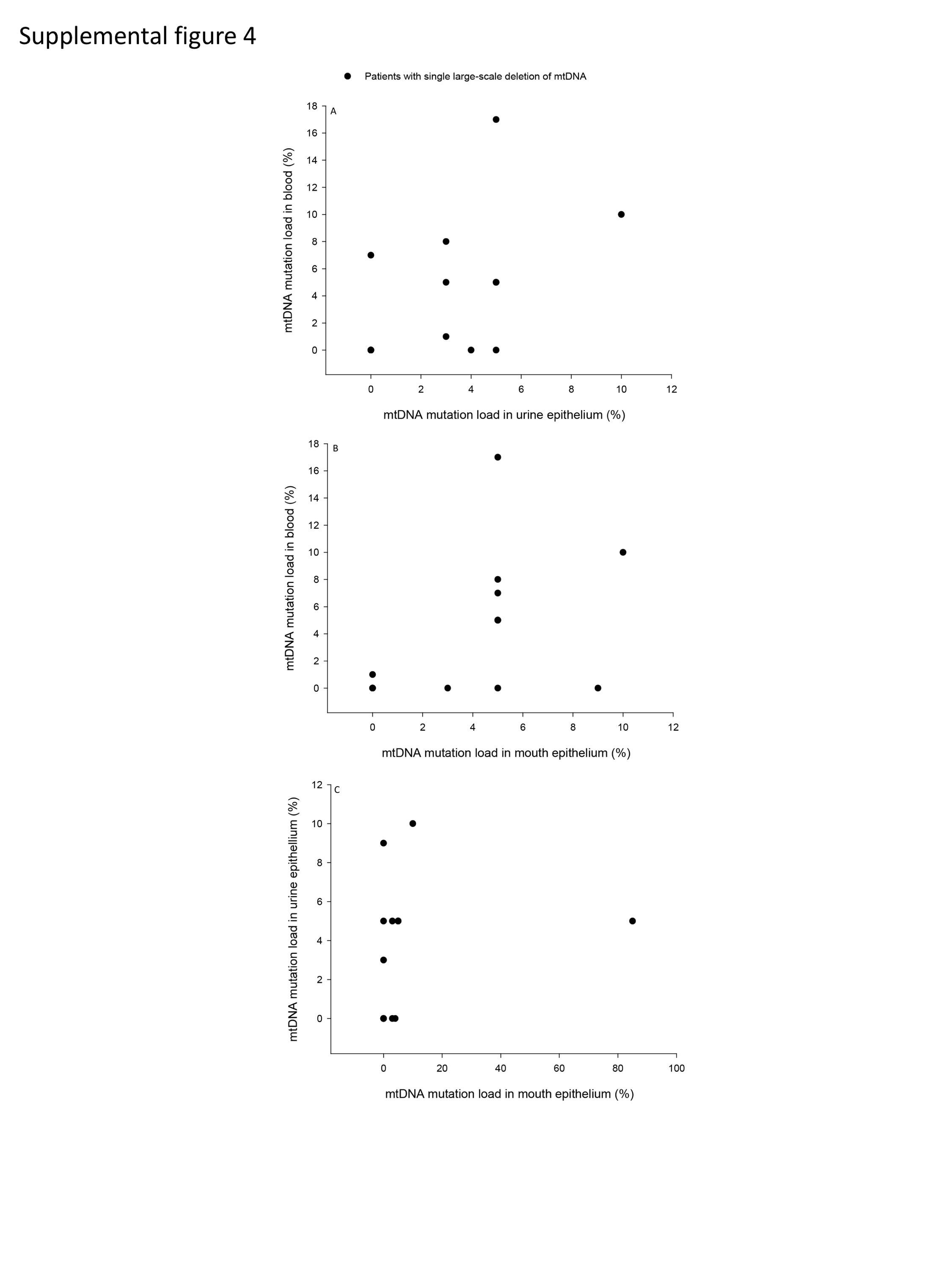

Supplement: Supplementary Figure 4 — Correlation between percentage mitochondrial DNA (mtDNA) mutation load in blood versus mtDNA mutation load in urine epithelium (A), percentage mitochondrial DNA (mtDNA) mutation load in blood versus mtDNA mutation load in mouth epithelium (B) and percentage mtDNA mutation load in urine epithelium versus mtDNA mutation load in mouth epithelium (C) in the individual 17 patients with single large-scale deletion of mtDNA. In graph (A) patient number #17 was left out in order not to have a ceiling effect and used as an example to show the difference from graph (C) where patient #17 has been left in. [file Image_4.jpg]

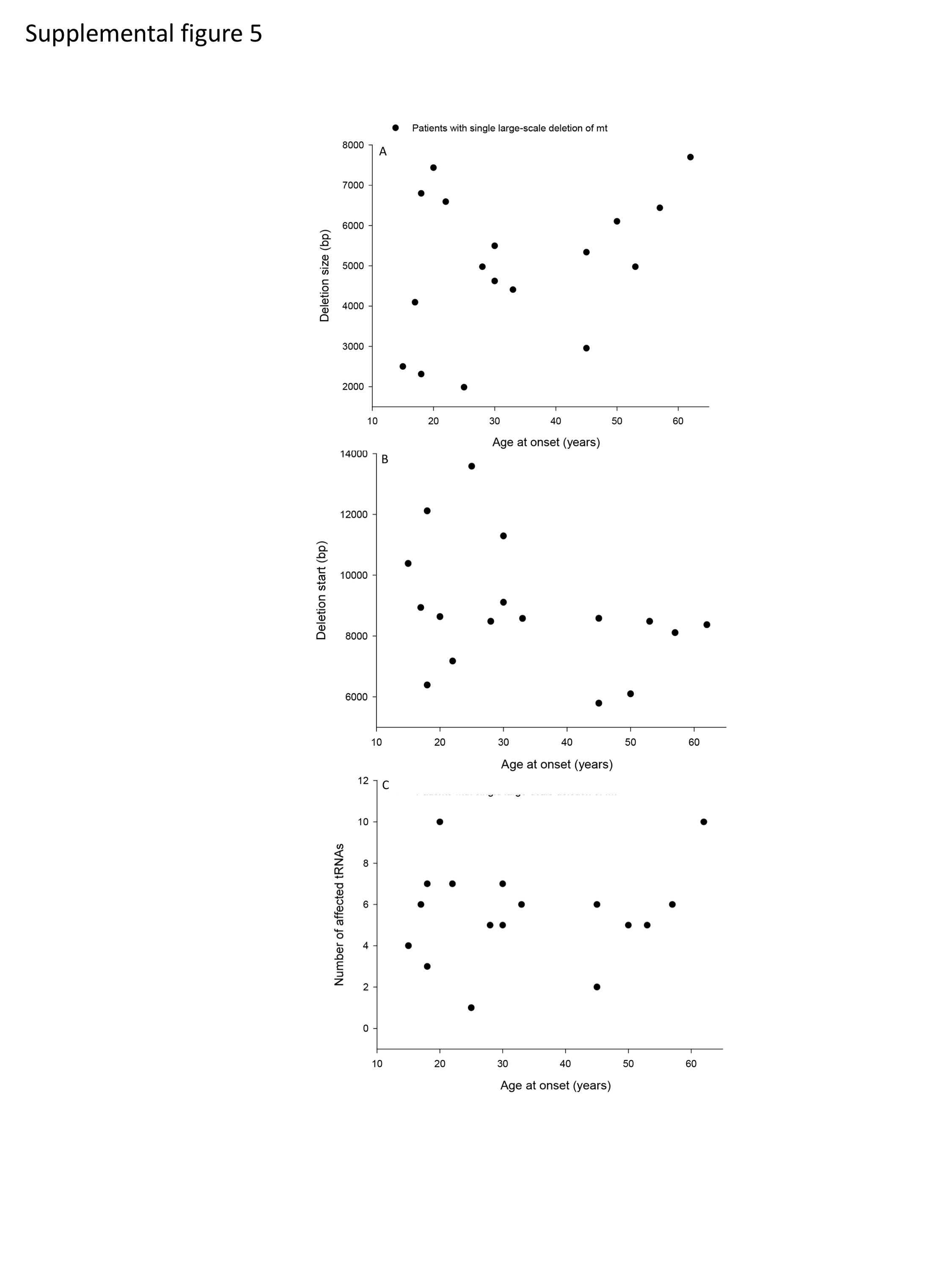

Supplement: Supplementary Figure 5 — Correlation between Age at onset versus deletion size (A), start of deletion (B) and number of tRNAs affected (C) in the individual 17 patients with single large-scale deletion of mtDNA. [file Image_5.JPEG]
